# Evolutionary Patterns in the Sequence and Structure of Transfer RNA: Early Origins of Archaea and Viruses

**DOI:** 10.1371/journal.pcbi.1000018

**Published:** 2008-03-07

**Authors:** Feng-Jie Sun, Gustavo Caetano-Anollés

**Affiliations:** Department of Crop Sciences, University of Illinois Urbana-Champaign, Urbana, Illinois, United States of America; University of California San Diego, United States of America

## Abstract

Transfer RNAs (tRNAs) are ancient molecules that are central to translation. Since they probably carry evolutionary signatures that were left behind when the living world diversified, we reconstructed phylogenies directly from the sequence and structure of tRNA using well-established phylogenetic methods. The trees placed tRNAs with long variable arms charging Sec, Tyr, Ser, and Leu consistently at the base of the rooted phylogenies, but failed to reveal groupings that would indicate clear evolutionary links to organismal origin or molecular functions. In order to uncover evolutionary patterns in the trees, we forced tRNAs into monophyletic groups using constraint analyses to generate timelines of organismal diversification and test competing evolutionary hypotheses. Remarkably, organismal timelines showed Archaea was the most ancestral superkingdom, followed by viruses, then superkingdoms Eukarya and Bacteria, in that order, supporting conclusions from recent phylogenomic studies of protein architecture. Strikingly, constraint analyses showed that the origin of viruses was not only ancient, but was linked to Archaea. Our findings have important implications. They support the notion that the archaeal lineage was very ancient, resulted in the first organismal divide, and predated diversification of tRNA function and specificity. Results are also consistent with the concept that viruses contributed to the development of the DNA replication machinery during the early diversification of the living world.

## Author Summary

The origins of the three major cellular lineages of life—Archaea, Bacteria, and Eukarya—and of viruses have been shrouded in mystery. In this study, we focus on transfer RNA, an ancient nucleic acid molecule that takes center stage in the process of protein biosynthesis and can be found everywhere in life. In a process that reconstructs history from molecular sequence and structure and at the same time forces molecules belonging to lineages into groups, we tested alternative hypotheses of origin and established when major organismal lineages appeared in evolution. Remarkably, timelines showed that Archaea was the most ancient lineage on earth and that viruses originated early in the archaeal lineage. Our findings unroot the universal tree of life, and, for the first time, provide evidence for an evolutionary origin of viruses.

## Introduction

Transfer RNA (tRNA) molecules are central to the entire translation process. They interact with the ribosomal RNA (rRNA) subunits as they are being ratcheted through the center of the ribosome [1],[2]. Their acceptor arms charge specific amino acids through the activity of cognate aminoacyl-tRNA synthetases, while triplets of bases on their ‘anticodon’ arms recognize complementary ‘codon’ sequences in messenger RNA. These and many other molecular interactions define the identities and functions of these tRNA adaptors and establish a genetic code that translates nucleic acid into protein information in the cell. The structural make-up of tRNA is therefore fundamental to our understanding of how the biosynthetic machinery was set up into place in an emerging protein and organismal world. tRNAs are clearly ancient molecules [3] and they have been used profusely to study the evolution of ancient life [4]–[8]. The identity and function of tRNAs are fundamentally delimited by the structure of the molecules, and structure is more conserved than sequence. In fact, we recently showed that tRNA structure carries deep phylogenetic signal and can be used directly to reconstruct evolutionary history [9]. However, understanding phylogenetic trees is challenging because tRNA evolution embeds a history of recruitment in which structures gain or co-opt new identities and functions or takeover established ones.

The hierarchical branching patterns of the universal tree of life portray the natural history of the living world. The current accepted universal tree proposes a tripartite world ruled by three superkingdoms, Archaea, Bacteria, and Eukarya [10]. This view stems fundamentally from the study of the small subunit of rRNA, a molecule that is also ancient and central to translation. The rise of evolutionary genomics with an analysis of entire repertoires of nucleic acid and protein molecules supports this tripartite scheme [11],[12]. However, the root of the universal tree remains controversial and so is the nature of the universal ancestor of all life that this root defines [13],[14]. We recently embarked on a systematic and global study of evolution of domain structure and organization in proteins [15],[16] (Wang and Caetano-Anollés, submitted). Structures were assigned to protein sequences in hundreds of completely sequenced genomes and a structural census of protein domains used to generate phylogenomic trees of protein architectures. The evolutionary genomic analysis defined a universal ancestor that was eukaryotic-like and had a relatively complex proteome [16]. It also established that the archaeal lineage was the most ancient and originated from reductive evolutionary tendencies in the use of protein architectures.

In order to explore if similar phylogenetic signatures were present in tRNA, we apply a well-established cladistic method [17],[18] that embeds structure directly into phylogenetic analysis [19]. The method involves identifying features characteristic of the secondary structure of RNA molecules, coding these features as linearly ordered multi-state characters, and using them to build phylogenetic trees with optimal tree search methods. The strategy has been used to reconstruct a tripartite tree of life from rRNA structure [17], trace evolution of rRNA in ribosomes [18], study the evolution of closely related phytopathogenic fungi [17] or distantly related members of the grass family [20], and explore the structural origin and evolution of retrotransposons in eukaryotes [21]. We also used the approach to study the evolution of the major structural and functional components of tRNA, establishing that tRNA molecules originated in the acceptor arm and providing further support to the ‘genomic tag’ hypothesis [9]. Here we reconstruct global phylogenetic trees using information embedded in both the sequence and structure of tRNA molecules. As we have shown previously (Sun and Caetano-Anollés, submitted), the intrinsically rooted trees revealed that tRNA with long variable arms (known as class II or type II tRNA) coding for amino acids Sec, Ser, Tyr, and Leu were ancient. However, trees failed to show clear patterns related to tRNA function, an observation that underscores the importance of recruitment and phylogenetic constraint (factors that restrict the acquisition of phenotypic traits or functions in lineages) in tRNA evolution. In order to sort out these confounding processes we built trees while forcing monophyletic groupings of taxa (sets that share a common ancestor) to test alterative hypotheses or establish evolutionary timelines of structural, functional, or organismal diversification. This strategy (known as constraint analysis in phylogenetics) provided an unanticipated window into early evolution of life.

## Results

**Figure 1:**
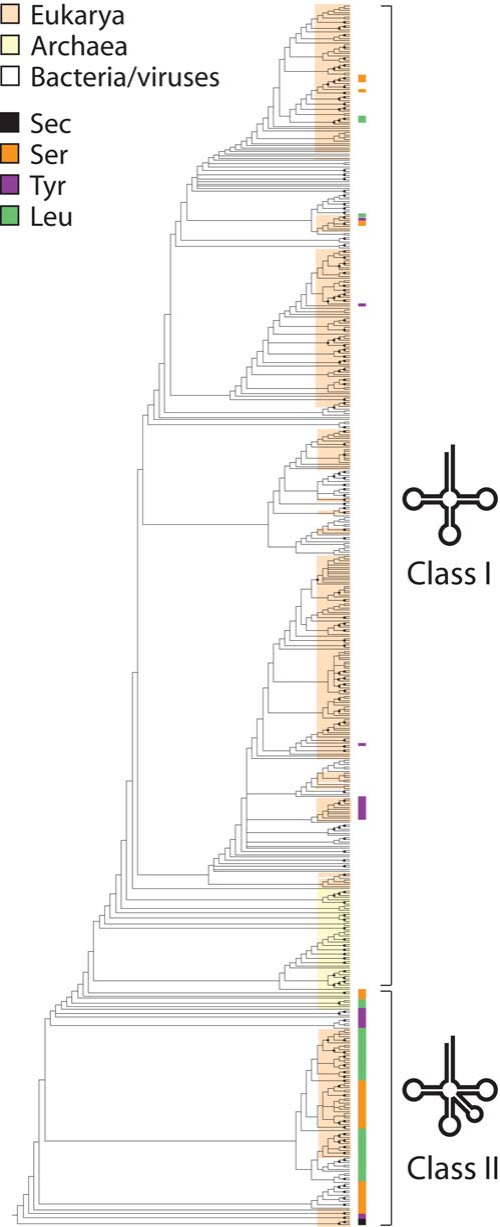
A global phylogenetic tree of tRNA molecules reconstructed from sequence and structure. MP analyses of data from 571 tRNA molecules resulted in the preset limit of 20,000 minimal length trees, each of 10,083 steps. Consistency index (CI) = 0.069 and 0.069, with and without uninformative characters, respectively; Retention index (RI) = 0.681; Rescaled consistency index (RC) = 0.047; g1 = −0.107. Terminal leaves are not labeled since they would not be legible. Nodes labeled with closed circles have BS values >50%. tRNA molecules belonging to different superkingdoms and viruses and coding for Sec, Ser, Tyr, and Leu are labeled with colors. Note several of these tRNAs have short variable arms and are derived in the tree.

Phylogenetic analyses of the combined dataset of sequence and structure of 571 tRNAs produced most parsimonious trees that were 10,083 steps in length and were intrinsically rooted (Figure 1). The tRNA set was obtained from Part 2 of the Bayreuth tRNA Database and represented organisms in the three superkingdoms of life and viruses and covered all isoacceptor variants and amino acid specificities (Table S1). This molecular set is unique since it contains information of modified bases and structures derived by comparative analysis (see Materials and Methods). Bootstrap support (BS) values were generally low for most clades (<50%), but this was generally expected given the large number of taxa (molecules) analyzed. Class II tRNA molecules with long variable arms, including tRNA^Sec^ and most tRNA^Ser^, tRNA^Tyr^, and tRNA^Leu^ isoacceptors, appeared at the base of the rooted trees (Figure 1). Besides this pattern, trees failed to reveal groupings that would indicate clear evolutionary links to organismal origin or molecular functions. The monophyly of tRNA belonging to each superkingdom (or viruses) or expressing different amino acid specificities was not revealed. Similarly, tRNAs with specificities for amino acids defined previously as being ancestral [22]–[27] did not form monophyletic groups. tRNA molecules sharing the first, second, or first and second bases in codons were not monophyletic either. These patterns were also observed in trees derived from partitioned matrices of superkingdoms or viruses (data not shown).

In order to uncover deep phylogenetic signals and test alternative evolutionary hypotheses we forced groups of tRNAs that shared a same organismal origin (molecules from each superkingdom of life or viruses) into monophyly using constraint analyses. We then recorded the length of the most parsimonious trees that were obtained and the number of additional steps (*S*) that were needed to force the constraint. This exercise was generally done with or without forcing class I and II tRNA molecules into separate groups, but overall results were congruent.

**Figure 2:**
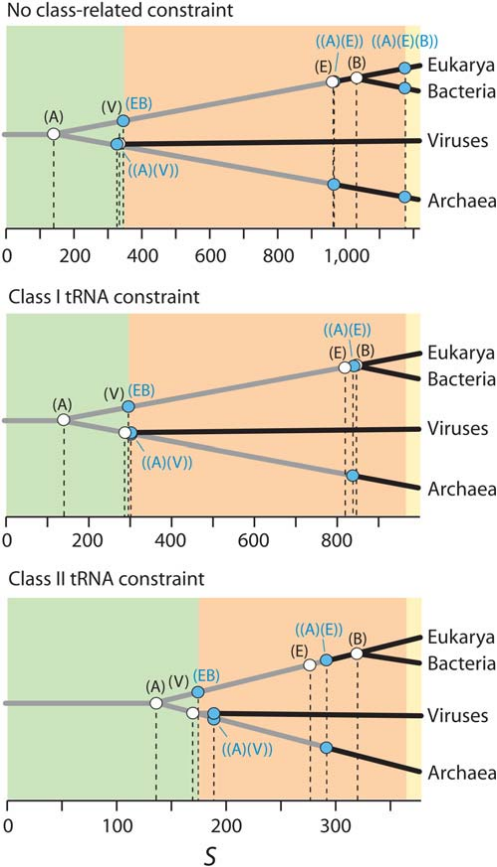
Timeline of organismal diversification. Constraints representing non-competing hypotheses of organismal relationship (white circles) are used to define a timeline for the appearance of lineages in a universal tree derived from the sequence and structure of tRNA. Blue circles represent constraints representing competing hypotheses. They illustrate both the most parsimonious lineage relationship and their coalescence. Areas colored in light green, salmon, and light yellow are delimited by lineage coalescence and describe three evolutionary epochs. The timeline is given in a scale of additional steps (*S*) needed to fulfill constraints. *S* values were not normalized.

Constraints related to the diversification of the organismal world (Table 1) consistently showed Archaea as the ancestral group (i.e., forcing archaeal tRNAs into monophyly was always associated with low *S*), followed by viruses, Eukarya, and Bacteria (with *S* increasing in that order) (Figure 2). Hypotheses of relationship among superkingdoms clarified further the possible rooting of the universal tree. Constraining molecules from Eukarya and Bacteria into a monophyletic group [constraint (EB)] was the most parsimonious solution and was consistent with an early split of two ancient lineages, one of archaeal origin and the other of eukaryal-bacterial origin. When forcing molecules from two of the three superkingdoms individually and as a group into monophyly, the Eukarya and Archaea dichotomy [constraint ((E)(A))] was most parsimonious. This suggests the earliest two superkingdoms to diversify were Eukarya and Archaea. The *S* values for these constraints indicated that their diversification always preceded the onset of Bacteria. Finally, constraining molecules from the three superkingdoms into three separate groups in all possible 3-taxon statements showed that a polytomous arrangement was the most parsimonious. *S* values exceeded those indicating the onset of Bacteria as a group. These patterns maintained when tRNA structural categories were constrained and all phylogenetic statements were congruent (Figure 2).

**Table 1:** Origins of the tripartite world

tRNA class	Organismal constraints	Test	*S*
Unconstrained	((A), B, E, V)	H	132
	((V), A, B, E)	H	336
	((E), A, B, V)	H	967
	((B), A, E, V)	H	1039
	((A, B, E), V)	H	339
	((B, E), A, V)	CH1	345
	((A, E), B, V)	CH1	971
	((A, B), E, V)	CH1	1038
	(((A)(E)), B, V)	CH2	966
	(((A)(B)), E, V)	CH2	1042
	(((B)(E)), A, V)	CH2	1164
	((A), (B), (E), V)	CH3	1171
	(((A)(B)(E)), V)	CH3	1171
	(((A)(B))(E), V)	CH3	1171
	(((A)(E))(B), V)	CH3	1178
	(((B)(E))(A), V)	CH3	1179
	((A), (B), (E), (V))	H	1190
Constrained	((Class I: A, B, E, V), (Class II: A, B, E, V))	H	232
	((Class I: A, B, E, V), (Class II: (A), B, E, V))	H	136
	((Class I: A, B, E, V), (Class II: (V), A, B, E))	H	168
	((Class I: A, B, E, V), (Class II: (B), A, E, V))	H	318
	((Class I: A, B, E, V), (Class II: (E), A, B, V))	H	276
	((Class I: A, B, E, V), (Class II: (B, E), A, V))	CH1	174
	((Class I: A, B, E, V), (Class II: (A, E), B, V))	CH1	296
	((Class I: A, B, E, V), (Class II: (A, B), E, V))	CH1	309
	((Class I: A, B, E, V), (Class II: ((A)(E)), B, V))	CH2	294
	((Class I: A, B, E, V), (Class II: ((A)(B)), E, V))	CH2	316
	((Class I: A, B, E, V), (Class II: ((B)(E)), A, V))	CH2	325
	((Class I: (A), B, E, V), (Class II: A, B, E, V))	H	143
	((Class I: (V), A, B, E), (Class II: A, B, E, V))	H	291
	((Class I: (E), A, B, V), (Class II: A, B, E, V))	H	814
	((Class I: (B), A, E, V), (Class II: A, B, E, V))	H	852
	((Class I: (B, E), A, V), (Class II: A, B, E, V))	CH1	297
	((Class I: (A, E), B, V), (Class II: A, B, E, V))	CH1	825
	((Class I: (A, B), E, V), (Class II: A, B, E, V))	CH1	851
	((Class I: ((A)(E)), B, V), (Class II: A, B, E, V))	CH2	843
	((Class I: ((A)(B)), E, V), (Class II: A, B, E, V))	CH2	870
	((Class I: ((B)(E)), A, V), (Class II: A, B, E, V))	CH2	961

The numbers of additional steps (*S*) required to force molecules into monophyly were calculated based on class (class I and II tRNAs) and organismal (three superkingdoms of life or viruses) constraints using MP analyses of combined tRNA structure and sequence data. The length of the most parsimonious trees derived from the combined data set was 10,083 steps. Each constrained group is given in parentheses and groups of tRNA molecules are indicated by superkingdoms of life (Archaea, Bacteria, and Eukarya) or viruses. Both chloroplast and mitochondria tRNAs were included in Bacteria. A = Archaea, B = Bacteria, E = Eukarya, V = viruses. CH, competing hypothesis; H, non-competing hypothesis.

We also explored the origins of viruses by constraining molecules from each individual superkingdom or viruses into monophyletic groups, together [e.g., (AV)] or separately [e.g., ((A)(V))] (Table 2). The most parsimonious scenario always linked the origins of viruses to the archaeal lineage, with *S* values matching those defining the organismal timeline (Figure 2). Dividing the viral sequences into two groups (i.e., viruses infecting Eukarya or Bacteria) maintained the linkage between the separated groups of viruses and Archaea for various competing hypotheses (Table 2).

**Table 2:** Origins of the viral world

tRNA category	Organismal constraints	Test	*S*
Unconstrained	((A, V), B, E)	CH1	342
	((B, V), A, E)	CH1	979
	((E, V), A, B)	CH1	1034
	(((A)(V)), B, E)	CH2	333
	(((B)(V)), A, E)	CH2	1164
	(((E)(V)), A, B)	CH2	1162
	((A, V_B_), V_E_, B, E)	CH3	249
	((B, V_B_), V_E_, A, E)	CH3	959
	((E, V_B_), V_E_, A, B)	CH3	1015
	((A, V_E_), V_B_, B, E)	CH4	198
	((B, V_E_), V_B_, A, E)	CH4	926
	((E, V_E_), V_B_, A, B)	CH4	955
	(((A)(V_B_)), V_E_, B, E)	CH5	246
	(((B)(V_B_)), V_E_, A, E)	CH5	1100
	(((E)(V_B_)), V_E_, A, B)	CH5	1088
	(((A)(V_E_)), V_B_, B, E)	CH6	192
	(((B)(V_E_)), V_B_, A, E)	CH6	1078
	(((E)(V_E_)), V_B_, A, B)	CH6	1018
Constrained	((Class I: A, B, E, V), (Class II: (A, V), B, E))	CH7	182
	((Class I: A, B, E, V), (Class II: (B, V), A, E))	CH7	289
	((Class I: A, B, E, V), (Class II: (E, V), A, B))	CH7	312
	((Class I: A, B, E, V), (Class II: ((A)(V)), B, E))	CH8	189
	((Class I: A, B, E, V), (Class II: ((B)(V)), A, E))	CH8	324
	((Class I: A, B, E, V), (Class II: ((E)(V)), A, B))	CH8	328
	((Class I: (A, V), B, E), (Class II: A, B, E, V))	CH9	292
	((Class I: (B, V), A, E), (Class II: A, B, E, V))	CH9	817
	((Class I: (E, V), A, B), (Class II: A, B, E, V))	CH9	855
	((Class I: ((A)(V)), B, E), (Class II: A, B, E, V))	CH10	301
	((Class I: ((B)(V)), A, E), (Class II: A, B, E, V))	CH10	971
	((Class I: ((E)(V)), A, B), (Class II: A, B, E, V))	CH10	961

The numbers of additional steps (*S*) required to force molecules into monophyly were calculated based on class (class I and II tRNAs) and organismal (three superkingdoms of life or viruses) constraints using MP analyses of combined tRNA structure and sequence data. The length of the most parsimonious trees derived from the combined data set was 10,083 steps. Each constrained group is given in parentheses and groups of tRNA molecules are indicated by superkingdoms of life (Archaea, Bacteria, and Eukarya) or viruses. Both chloroplast and mitochondria tRNAs were included in Bacteria. A = Archaea, B = Bacteria, E = Eukarya, V = viruses, V_B_ = viruses associated with Bacteria, V_E_ = viruses associated with Eukarya. CH, competing hypothesis.

Finally, we constrained trees according to isoacceptor group and then according to organismal group, or vice versa, with or without constraining tRNA categories (Table 3). A scenario in which organismal (superkingdom) diversification predated tRNA functional divergence was always more parsimonious (*S* = 2,338–2,481) than one where functional divergence predated organismal diversification (*S* = 2,415–2,534).

**Table 3:** The numbers of additional steps (*S*) required to force molecules into monophyly based on tRNA category, amino acid specificity, and organismal constraints using MP analyses of combined tRNA structure and sequence data

tRNA category	Constraints	*S*
Unconstrained	Superkingdom diversification prior to functional divergence: ((A: (Ala), (Arg), …, (Sec)), (B: (Ala), (Arg), …, (Sec)), (E: (Ala), (Arg), …, (Sec)), (V: (Ala), (Arg), …, (Sec)))	2481
	Functional divergence prior to superkingdom diversification: ((Ala: (A)(B)(E)(V)), (Arg: (A)(B)(E)(V)), …, (Val: (A)(B)(E)(V)))	2534
Constrained	Superkingdom diversification prior to functional divergence: ((Class II: (A: (Ser)(Sec)(Leu)(Tyr)), (B: (Ser)(Sec)(Leu)(Tyr)), (E: (Ser)(Sec)(Leu)(Tyr)), (V: (Ser)(Sec)(Leu)(Tyr))), (Class I: (A: (Ala), (Arg), …, (Sec)), (B: (Ala), (Arg), …, (Sec)), (E: (Ala), (Arg), …, (Sec)), (V: (Ala), (Arg), …, (Sec))))	2338
	Functional divergence prior to superkingdom diversification: ((Class II: (Ser: (A)(B)(E)(V)), (Sec: (A)(B)(E)(V)), (Leu; (A)(B)(E)(V)), (Tyr: (A)(B)(E)(V))), (Class I: (Ala: (A)(B)(E)(V)), (Arg: (A)(B)(E)(V)), …, (Val: (A)(B)(E)(V))))	2415

The length of the most parsimonious trees derived from the combined data set was 10,083 steps. Each constrained group is given in parentheses. Both chloroplast and mitochondria tRNAs were included in Bacteria. A = Archaea, B = Bacteria, E = Eukarya, V = viruses. Amino acids are indicated by the International Union of Pure and Applied Chemistry (IUPAC) 3-letter nomenclature.

Since constraint analyses could be biased by unequal rates of evolution, we calculated average number of character change per branch in consensus trees generated from partitioned data matrices (Table 4). An analysis of variance (ANOVA) showed values were not significantly different in the three superkindoms of life and viruses (*p*>0.05). Similarly, we did not find differences when random trees were compared (not shown).

**Table 4:** Rates of evolution in the three superkingdoms and viruses derived from strict consensus trees

	Assigned branch length	Minimum length	Maximum length
Archaea (59 leaves)	5.25±6.44 (425)	4.78±5.80 (387)	5.74±6.57 (465)
Bacteria (275 leaves)	4.20±5.34 (1,776)	3.97±5.20 (1,679)	4.45±5.44 (1,881)
Eukarya (220 leaves)	5.19±6.16 (1,667)	4.81±6.02 (1,544)	5.60±6.34 (1,796)
Viruses (17 leaves)	5.85±9.87 (193)	5.42±9.43 (179)	6.27±9.89 (207)

The average number of character changes per branch (±standard deviations) are listed for assigned, minimum, and maximum values. The total numbers of character changes in the trees are given in parentheses. ANOVA showed average branch lengths were not significantly different between different superkingdoms or viruses (assigned branch lengths, df: 3, 854; F = 2.

## Discussion

### Deep evolutionary patterns embedded in tRNA phylogenies

In order to uncover evolutionary patterns related to organismal diversification, we first generated rooted phylogenetic trees using information embedded in the structure and sequence of tRNA (Figure 1). As expected, class II tRNA molecules with long variable arms coding for Sec, Ser, Tyr, and Leu appeared at the base of the rooted trees and were ancient. We also observed a rather tight paraphyletic clustering of tRNAs of archaeal origin. However, we were unable to reveal any other pattern of significance in the trees; no monophyletic groupings could be established when tracing tRNA function, codon identity, or organismal origin (data not shown). In order to untangle the intricate history of tRNA, we forced trees to acquire pre-defined tree topologies representing competing (alternative) or non-competing phylogenetic hypotheses, constrained the exploration of tree space during phylogenetic searches, and produced sub-optimal tree reconstructions. Competing hypotheses were contrasted and those that imposed a minimum number of additional steps (*S*) on the optimal tree (i.e., more parsimonious) were not rejected. Using this approach, we tested for example competing chronologies or sister taxa relationships related to organismal diversification. In turn, non-competing hypotheses were ranked by the values of *S* according to some external evolutionary model. In this study, they were used to define timelines of first appearance of superkingdoms and viruses in evolution. Hypotheses of origin that were satisfied with fewer steps were considered less affected by the confounding effects of recruitment in lineages and more ancient than those that required more steps. In other words, it was easy to merge lineages in backwards time (a process known as coalescence) to fit the constraint. Plots mapping the correlation between *S* and number of nodes from a hypothetical tRNA ancestor in the trees confirmed the validity of this assumption of ‘polarization’ (data not shown). This type of analysis is not new. In cybernetics it is known as ‘constraint analysis’ and represents a formal method of decomposing a reconstructable system into its components by imposing natural or man-made limitations [28]. The method is widely used in cladistic and phylogenetic analyses to test for example hypotheses of monophyly [29], but to our knowledge, has never been used to dissect systematically patterns in a phylogenetic tree.

Two fundamental assumptions support the analysis. First, we assume tRNA structures acquired new identities and functions as the genetic code expanded, and that different structures were co-opted for the task in different lineages and different functional contexts. This assumption seems reasonable. Recruitment processes are common in evolution of macromolecules. In cellular metabolism, for example, enzymes are often recruited into different pathways to perform new enzymatic functions [16],[30],[31]. Moreover, structural diversification of tRNA appeared to have predated organismal diversification [32] (Sun and Caetano-Anollés, submitted) and the functions and identities attached to present-day tRNA structures probably developed in lineages and were shuffled by horizontal gene transfer. Second, we assume old tRNA structures developed or recruited new functions (co-options) more often than new tRNA structures acquired old functions (takeovers). This assumption is also reasonable and appears to be supported by our studies of enzyme recruitment in metabolism (Kim et al., ms. in preparation). Our trees show several instances of takeovers, in which modern class I structures lacking the long variable arms took over ancient amino acid charging functions associated with class II structures (Figure 1; Sun and Caetano-Anollés, submitted). However, old structures have more chances to succeed in a diversifying world, as they spread through lineages. Younger structures in turn are restricted to the lineage in which they originated, and can only spread further through horizontal transfer events. One implication of this assumption is that older functions will be less prone to co-options than younger functions, and that tRNA belonging to older lineages will be less affected by co-options than those in younger lineages. Consequently, ancient molecules sharing functions or belonging to selected lineages will be more easily constrained than younger variants in phylogenetic reconstruction.

We also assume phylogenies are free from systematic errors and the confounding effects of mutational saturation, long branch attraction artifacts, and unequal rates of evolution along branches of the trees [11]. However, most branching events in these phylogenies happened a relatively long time ago and phylogenetic analyses of ancient molecules carry all the problems of deep reconstruction [33]. While the impact of some of these effects diminishes when using multi-state characters in tRNA structure [34],[35], different rates of change could alter the coalescense of lineages and our results. For example, increased rates of change known to occur in rapidly evolving viral molecules could increase expected *S* values, making the viral lineage artificially younger. Nevertheless, an analysis of rates of change in consensus and random trees derived from partitioned data matrices showed that evolutionary rates of tRNAs in the three superkingdoms of life or viruses were not significantly different in our analysis (Table 4). The fact that evolutionary rates in the four lineages were similar decreases the impact of unequal rates of evolution and underscores the conserved nature of tRNA structure when compared to sequence. Similarly, problems of statistical consistency related to long branch attraction could bias the reconstruction of the tRNA tree. These artifacts, which are rather common in sequence analysis, result from unequal rates of variation in branches and the interplay of short and long branches in a tree [36]. They are however not so much related to branch length (which in our analyses do not vary considerably; Table 4) but to changes of a same character state occurring preferentially in long branches, forcing the tree-building method to join them artificially. However, the probability of these covarying homoplasies is known to decrease with increases in character states, as with the multi-state structural characters of this study [34], and when branches are separated by increased taxon sampling [37]–[39]. Consequently, large trees as the tRNA trees we have reconstructed from sequence and structure in this study should be considerably less prone to consistency problems [38],[39] than the four-taxon statements related to sequences originally used to define them [36], especially if they involve multiple character states depicting structure.

### Timelines of organismal diversification and the birth of the tripartite world

We constrained tRNA groups according to organismal origin using different schemes and tested possible competing and non-competing hypotheses describing timelines of organismal diversification and possible topologies of the universal tree of life (Figure 2). Constraining tRNAs belonging to individual superkingdoms or viruses showed Archaea as the most ancestral group, followed by viruses, Eukarya, and Bacteria, in that order. This timeline already suggests a very early split of the archaeal lineage in evolution. An analysis of the three possible two-superkingdoms single-group constraints showed that forcing molecules from Eukarya and Bacteria into a single monophyletic group [constraint (EB)] was most parsimonious and confirmed the early split of lineages and separation of Archaea. It also suggested an important lineage relationship between Eukarya and Bacteria and a relative time frame for their coalescence as a group. Interestingly, *S* values for the eukaryal-bacterial lineage always coincided with those for the viral group, suggesting viruses appeared at a time when this early lineage was coalescing (see below). Forcing molecules belonging to two superkingdoms into separate monophyletic groups once again confirmed the early split of Archaea and the late onset of Eukarya; the most parsimonious solution [constraint ((A)(E))] showed that the separate coalescence of the archaeal and eukaryal lineages followed the appearance of Eukarya as an organismal group [constraint (E)] and always preceded the appearance of Bacteria [constraint (B)]. Finally, constraining the three superkingdoms into separate monophyletic groups resulted as expected in higher *S* values, reflecting the coalescence of all lineages of a fully diversified organismal world. Out of all possible competing hypotheses of relationship several alternatives were most parsimonious, including an unresolved 3-taxon statement [constraint ((A)(B)(E))]. The confounding effects of recruitment were probably severe and were incapable of revealing the root of the universal tree at these high *S* values and late evolutionary stages.

The timeline of organismal diversification provides evidence that the lineage of Archaea segregated from an ancient community of ancestral organisms and established the first organismal divide. The scenario of organismal diversification described above is congruent with our recent phylogenomic analyses of protein structure [16] and domain organization (Wang and Caetano-Anollés, submitted) in hundreds of completely sequenced genomes. The result is also congruent with recent studies that have used tRNA paralog (alloacceptor) clustering as a measure of ancestry of tRNA genotypes [40] and multiple lines of evidence [41],[42] to suggest a *Methanopyrus*-proximal root of life. Although it is popularly accepted that the universal tree of life based on molecular phylogenies is rooted in the prokaryotes and that Archaea and Eukarya are sister groups, these recent results together with those presented in this paper offer compelling arguments in favor of an early appearance of the Archaea.

Our evolutionary timeline is also remarkable in that it identifies three epochs in the evolution of the organismal world that were analogous to those proposed earlier [16]: (1) an *architectural diversification* epoch in which tRNA molecules diversified their structural repertoires (light green areas in Figure 2), (2) a *superkingdom specification* epoch in which tRNA molecules sorted in emerging lineages that specified superkingdoms Archaea, Bacteria, and Eukarya (salmon areas), and (3) an *organismal diversification* epoch that started when all tRNA coalesced in each superkingdom (light yellow areas).

The evolutionary patterns observed in timelines appeared consistently in the absence or presence of class I or class II tRNA structural constraints (Figure 2). This suggests structural diversification predated organismal diversification during evolution of tRNA. Similarly, a scenario in which organismal diversification predated amino acid charging diversification was more parsimonious (Table 3), suggesting the discovery of both amino acid charging and associated codon function occurred in expanding lineages. These conclusions are supported by a recent study that compared sequence matches between tRNA halves and suggested the modern tRNA cloverleaf arose prior to the divergence of modern tRNA specificities and the three superkingdoms of life [32].

### The early evolutionary appearance of viruses

The organismal timeline inferred from tRNA sequence and structure showed Archaea was the most ancient superkingdom but established that viruses were also ancient. Viruses are relatively simple living entities and in many cases maintain a regular structure. They have long been considered fragments of cellular genomes and not living organisms and were generally excluded from consideration in evolutionary scenarios of the tripartite world, despite being important components of the biosphere. The importance of viruses and their potential roles in early cellular evolution were recently reevaluated [43]. A comparative analysis of structure and function, including virion assembly principles, suggested both RNA and DNA viruses may have been more ancient than previously thought, possibly even more ancient than the common ancestor of life [43]. However, they probably had a polyphyletic origin because structurally and functionally related viruses infect hosts in different lineages and even in different superkingdoms of the universal tree [44],[45]. It is therefore possible that viruses form lineages and share a common ancestor, and that these lineages extend from the root to all branches in the tree of life. For example, the overall similarity of viral structures, such as coat protein folds enclosing nucleoprotein filaments, suggests a common mechanism for their appearance. The construction of phylogenies addressing the questions of origin and evolution of viruses in the context of the three superkingdoms are now possible with the increasing number of sequenced genomes of viral origin. In fact, comparative genomic analyses suggested viruses could be the source of new proteins for cells [46]. Many DNA informational proteins encoded today in cellular genomes probably originated in the viral world and were later transferred into the three cellular superkingdoms. Forterre recently proposed that DNA itself appeared in ancestral viral lineages [47],[48]. He later on extended this proposal by suggesting that the DNA replication machineries of each superkingdom originated from three different ancestral viral lineages [49]. In his latest proposal, each cellular superkingdom originated independently from the fusion of an RNA-based cell and a large DNA virus [50].

In order to establish if the origin of the viruses was linked to one or more of the three superkingdoms of life we constrained viral and individual superkingdom tRNAs into competing monophyletic relationships (Table 3). Remarkably, most parsimonious constraints indicated viruses that associate with Eukarya and Bacteria had an origin in the archaeal lineage (Figure 2). The origin of viruses in Archaea is remarkable, especially if one considers the exceptional diversity and morphotype complexity of archaeal viruses [51]. Such an origin is compatible with the proposal by Forterre and colleagues that the transition from RNA to DNA genomes occurred in the viral world, and that cellular DNA and its replication machineries originated via transfers from DNA viruses to RNA cells. In fact, our phylogenomic analysis of structure [16] suggests a substantial portion of the replication machinery was developed during the architectural diversification phase immediately after reductive tendencies were already set in the archaeal lineage. This coincides with the relative time of emergence of viruses in the ancient world that was derived in this study. Since the appearance of a molecularly complex universal ancestor preceded the appearance of viruses, our results remain compatible with the accepted view that viruses originated from fragments of genetic material that escaped from the control of the cell and became parasitic (the escape theory) [52]–[55].

The origin of viruses is generally complex and may involve more than one mechanism [56]. Although several major classes of viruses are monophyletic, a common viral ancestry has not been evident [57]. Sequence analysis of viral genomes with various lengths (ranging from a few to hundreds of kilobases and containing several to hundreds of genes) and types (ranging from double-stranded DNA to single-stranded RNA) failed to reveal a common origin, suggesting instead polyphyletic (multiple) origins. However, a focus on sequence alone could be misleading. The viruses as a group contain more structural genomic diversity than cellular organisms such as plants, animals, or bacteria put together, and their sequences are fast evolving [58]. This could erase deep evolutionary history and confound analysis. Moreover, viruses also share many common features (e.g., genes coding for key proteins involved in viral replication and morphogenesis, parasitic nature of the replication mechanisms) not shared by any kind of cellular organisms [57], and these could be used to claim monophyly. This is especially true if the proposed ancient viral world existed [57]. This world harbored viral genes that retained their identity throughout the entire history of life. By this definition, the primordial pool of primitive genetic elements would be the ancestors of modern cellular and viral genes. This means that most, if not all, modern viruses were derived from elements that belonged to the primordial genetic pool, perhaps representing primitive form of self replicating DNA and precursor of life [59].

We end by noting that due to the small number of viral sequences sampled in our study, the conclusions drawn here should be taken with caution. However, a separate undergoing study analyzing a comprehensive dataset of tRNA sequences and structures but lacking information on base modifications support the evolutionary patterns presented in this study (Ospina, Sun, and Caetano-Anollés, unpublished).

## Materials and Methods

### Data

Part 2 (compilation of tRNA sequences) of the Bayreuth tRNA Database (http://www.staff.uni-bayreuth.de/btc914/search/index.html; September 2004 edition; Table S1) contains a total of 571 tRNA sequences at RNA level with cloverleaf secondary structures. The structures were derived by comparative analysis using an alignment that is most compatible with tRNA phylogenies and known 3-dimensional models of structure [60],[61]. The composition of part 2 was not pruned in our analyses and represents the most complete tRNA dataset currently available that contains information about base modifications. A total of 42 structural characters describing geometrical features of tRNA molecules (Table S2) were scored, establishing character homology by the relative position of substructures in the cloverleaf [9] (Sun and Caetano-Anollés, submitted). The length (the total number of bases or base pairs) and number of the substructures were coded as character states and were defined in alphanumerical format with numbers from 0 to 9 and letters from A to F. The minimum state (0) was given to missing substructures. We followed the Bayreuth database to treat the modified bases as deviations from the cloverleaf model. They were not allowed to establish canonical Watson-Crick pairs. Each helical stem region was scored as two complementary sequences (5′ and 3′ sides). The dataset was then partitioned into four subsets categorized by molecules belonging to each of the three superkingdoms or viruses/bacteriophages. In this study, a “total evidence” approach [62],[63] (also called “simultaneous analysis” [64]) was invoked in phylogenetic analysis to combine both sequence and structure data of the complete (571 tRNAs) and partitioned matrices. The goal of this analysis was to provide stronger support for the phylogenetic groupings recovered from analyses of structural data.

### Phylogenetic analysis

We treated structural features in molecules as phylogenetic multi-state characters with character states transforming according to linearly ordered and reversible pathways. Character state transformations were polarized by assuming an evolutionary tendency towards molecular order. Characters were analyzed using maximum parsimony (MP), a popular phylogenetic optimization method that searches for solutions that require the least amount of change. It is appropriate to treat geometrical features as linearly ordered characters because RNA structures change in discrete manner by addition or removal of nucleotide units. This causes gradual extension or contraction of geometrical features. Although insertion and deletion are also possible, they are more costly. The validity of character argumentation has been discussed in detail elsewhere [9],[17],[18],[20]. A considerable body of evidence supports our polarization hypothesis depicting generalized trends applied to the structure of molecules: (i) the study of extant and randomized sequences shows that evolution enhances conformational order and diminishes conflicting molecular interactions over those intrinsically acquired by self-organization [20], [65]–[70], (ii) a molecular tendency towards order and stability has been experimentally verified using thermodynamic principles generalized to account for non-equilibrium conditions [71]; (iii) a large body of theoretical evidence supports the structural repertoire of evolving sequences from energetic and kinetic perspectives [72]–[74], with some important predictions confirmed experimentally [75], (iv) phylogenies generated using geometrical and statistical structural characters are congruent [9],[20],[21], and (v) the reconstructions of rooted trees generated from sequence, structure, and genomic rearrangements at different taxonomical levels are congruent [17], [18], [20], [21], [76]–[78]. Phylogenetic trees were polarized by distinguishing ancestral states as those thermodynamically more stable. This results in reversible character transformation sequences that are directional and show asymmetry between gains and losses. Maximum and minimum character states were defined as the ancestral states for structures that stabilize (stems, modified bases, and G:U base pairs) and destabilize tRNAs (bulges, hairpin loops, and other unpaired regions), respectively.

All data matrices were analyzed using equally weighted MP as the optimality criterion in PAUP* v. 4.0 [79]. Because MP may outperform maximum likelihood (ML) approaches [34],[35], the use of MP is particularly appropriate for our analysis. ML is precisely MP when character changes occur with equal probability but rates vary freely between characters in each branch and when using large multi-step character state spaces (decreasing the likelihood of revisiting a same character state on the underlying tree). This makes MP statistically consistent. Reconstructions of MP trees were sought using heuristic search strategies; 1,000 heuristic searches were initiated using random addition starting taxa, with tree bisection reconnection (TBR) branch swapping and the MulTrees option selected. One shortest tree was saved from each search. Hypothetical ancestors were included in the searches for the most parsimonious trees using the Ancstates command. BS values [80] were calculated from 10^5^ replicate analyses using “fast” stepwise addition of taxa in PAUP*. The g_1_ statistic of skewed tree length distribution calculated from 10^4^ random parsimony trees was used to assess the amount of nonrandom structure in the data [81].

### Constraint analysis

Constraint analysis restricts the search of optimal trees to pre-specified tree topologies defining specific monophyletic groups, and was used here to test alternative or compare non-mutually exclusive hypotheses. The number of additional steps (*S*) required to force (constrain) particular taxa into a monophyletic group was examined using the “enforce topological constraint” option of PAUP*. The additional steps define an evolutionary distance that can be use to test alternative phylogenetic hypotheses or to compare hypotheses that are not mutually exclusive. The latter approach was used to construct evolutionary timelines, in which lower *S* values corresponded to ancient tRNAs, a trend that was derived from the rooted trees (and embedded assumptions of polarization). Constraint analyses were conducted based on amino acid specificity or grouping of molecules by organismal superkingdoms or viruses.

## Supporting Information

Table S1 Taxonomic distributions of the 571 tRNA molecules examined in the phylogenetic study.
(0.04 MB DOC)

Table S2 Structural characters and their statistics (range and mean ± standard deviation) used in the phylogenetic analyses of 571 tRNA molecules.
(0.10 MB DOC)
